# Aluminum and copper oxide nanoparticles alter secondary metabolites, antioxidants, and biochemical markers in key lime: *in vitro* approach

**DOI:** 10.1186/s12870-025-07802-1

**Published:** 2025-12-15

**Authors:** Marwa T. El-Mahdy, Eman Abdelazez Abulfadl, Mona F.A. Dawood

**Affiliations:** 1 Department of Pomology, Faculty of Agriculture, Assiut University, Assiut, 71526 Egypt; 2 Olive and semi-arid Zone Fruits Research Department, Horticulture Research Institute, Agricultural Research Center, Giza, 12619 Egypt; 3 Department of Botany and Microbiology, Faculty of Science, Assiut University, Assiut, 71516 Egypt

**Keywords:** Al_2_O_3_-NPs, CuO-NPs, Citrus aurantifolia, Secondary metabolites, Chelation agents, Ionomics

## Abstract

**Background:**

The expansive usage of metal oxide nanoparticles (NPs) as a potential constituent in modern nano-enabled products raises threats to environmental safety and crop production. Key lime is an economic plant enriched with secondary metabolites and imperative bioactive compounds. NPs can alter lime’s essential components, making it an ideal model for monitoring NPs’ toxicity. Hence, a comparative analysis of two synthesized metallic NPs, aluminum (Al_2_O_3_) and copper (CuO) at concentrations of 0, 100, 200, and 500 mg/L, was conducted to assess their impact on the growth and quality attributes of lime plants under controlled micro-conditions.

**Results:**

Observations from germination, growth, physiological, biochemical, and ionomic attributes showed that Al_2_O_3_-NPs had variable effects in a concentration-dependent manner, while CuO-NPs were toxic at all concentrations. Heatmap and principal component analysis revealed that CuO-NPs instigated more pronounced toxic effects compared with Al_2_O_3_-NPs at each applied concentration, as evidenced by heightened oxidative stress symptoms. CuO-NPs exerted their toxicity by over-accumulating reactive oxygen radicals and methylglyoxal and elevating lipoxygenase activity, causing peroxidation of membrane lipids and attenuation of photosynthetic pigments. Al_2_O_3_-NPs-treated plants relatively up-regulated the pool of phenolics, flavonoids, anthocyanins, ascorbic acid, and α-tocopherol as well as stimulated the activity of enzymatic antioxidants. Ionomics analysis showed excessive copper accumulation in toxic levels (~ 15-fold), which triggered nutritional imbalance (Mg, Si, P, S, Cl, K, Ca, Fe, Cu, and Zn) and disruption of chelating molecules, nitric oxide, and hydrogen sulfide.

**Conclusions:**

This work provides precious insights into the differential impacts of metallic NPs on the development and quality of lime plants and the underlying mechanisms involved in NPs accumulation, highlighting the possible hazards of manufactured NPs in the environment, especially for valuable plant species. However, the controlled use of NPs—particularly aluminum oxide—may offer agronomic benefits, but their application must be carefully handled to prevent toxicity.

## Introduction

In the current era, the technology of manipulating tiny structures of materials (1–100 nm), which is known as nanotechnology, is one of the most modern applications in many disciplines of science and is rapidly unveiling for new purposes. Nowadays, nanoparticles (NPs) have emerged in hundreds of industrial and agricultural applications such as agrochemicals, fertilizers, and pesticides, that exhibit extraordinary profits [1, 2]. However, alongside these theoretical and practical advances, the rampant usage of nanomaterials increases the likelihood of their release into the biota, posing substantial harmful effects on plant growth [3]. Hence, risk assessment studies should be considered, and it is crucial to elucidate the mode of interaction between NPs and plants to analyze scenario-oriented nanotechnology applications in agricultural systems.

Aluminum oxide nanoparticles (Al_2_O_3_-NPs) and copper oxide nanoparticles (CuO-NPs) are known as two typical types of metallic NPs that are burgeoningly used in high-technology industries according to their catalytic, magnetic, and moldability features [4]. In the agricultural sector, Al_2_O_3_-NPs [5] and CuO-NPs [6] have been recently used as nano-fertilizers to deliver nutrients and boost crop yields. They are effectively implemented in pesticides and fungicides. CuO-NPs, for example, have been applied for plant protection against fungal infection in roses [7]. However, more than 90% of the used pesticides are washed out during application [8]. Therefore, the current situation suggests that large portions of metallic NPs prevail either in the aquatic or terrestrial ecosystems.

Simultaneously, an increasing body of literature have depicted that metallic NPs display toxicity to plants in multiple ways, which can decline crop quantity and quality. Exposure to Al_2_O_3_-NPs caused a disturbance of the metabolic activities and physiological processes in tobacco [9], tomato [10], barley [11], and maize [12], and induced changes in the secondary metabolites of basil [13]. CuO-NPs at low concentrations (20 mg/L) stimulated the production of morphinan alkaloids in *Papaver orientale* L., while at higher concentrations (40 mg/L), they increased H_2_O_2_ production and decreased alkaloid measurements [14]. Presumably, the complex interplay of various factors such as the properties of NPs (i.e., type, dose, and size), exposure time, crop family, soil traits, etc., could exert positive or toxic impacts on plants [15–17]. As high amounts of metallic NPs can leach into soil compartments and surrounding environments, metallic NPs are considered potentially hazardous pollutants. In this scene, noxious NPs would infiltrate the human body through food chains and phytomedicines derived from medicinal plants that thrive in NPs-polluted sites, exposing living systems to acute health risks. Hence, it is essential to analyze the possible effects of metallic NPs on plants, more so to guarantee the security of human food and medicine.

Despite the stimulative benefits of metallic NPs, there is little information on the uptake and concentration of metallic NPs in plant tissues that contribute to their deleterious effects, particularly concerning the medicinal plants or the plants employed for the production of biologically active compounds. Clarifying the mechanisms underlying the interactions between NPs and medicinal plants is fundamental, as it is integral for contemporary agricultural practices and ecological balance. Key lime (*Citrus aurantifolia*, Rutaceae family) is an important fruit crop with boundless medicinal properties. Lime is highly regarded as a promising source of secondary metabolites and therapeutic characteristics due to its antibacterial, anticancer, and antioxidant features [18, 19]. In comparison to all citrus fruits, key lime affords the highest quantities of eriocitrin, which has been exhibited to retain the greatest antioxidant capacity among all other glycoside flavonoids [20]. Since the COVID-19 pandemic, citrus species have attracted special attention due to their high content of flavonoids and phenolic compounds, which contribute to prompting the human immune system, qualifying lime to be involved in pharmaceutical and industrial drug products [21]. Notably, the annual output of lime declined by 6.9% in 2019–2020 compared to 2018− 2019 due to numerous threatening factors [22]; however, the toxicity of metallic NPs on citrus species, including key lime, is seldom studied. In this trial, Al_2_O_3_-NPs and CuO-NPs were implemented in the in vitro growth media of lime at different concentrations. Through the study, the impact of Al_2_O_3_-NPs and CuO-NPs on growth, secondary metabolites, antioxidant capacity, and biochemical traits was assessed. This framework has important implications for understanding the interaction between metallic NPs and plants with medicinal properties and advancing our knowledge about the environmental risks of metallic NPs.

## Materials and methods

### Synthesis and characterization of Al_2_O_3_-NPs and CuO-NPs

#### Synthesis of Al_2_O_3_-NPs by hydrothermal method

The standard synthesis technique involved dissolving aluminum sulfate (Al_2_(SO_4_)_3_.18H_2_O 98% purity) and polyethylene glycol in 100 mL distilled water (DW) as described by Rabu et al. [23]. Next, the liquid was incubated at room temperature and under stirring for 60 min with a magnetic stirrer. Ammonia (25%) was mixed with the previous solution, which developed a milky precipitate at pH 9. The solution was incubated in an autoclave (140 °C for 48 h). The final residue was dehydrated at 100 °C for 1 h and calcined at 600 °C for 6 h.

#### Synthesis of CuO-NPs by co-precipitation method

Cupric nitrate (0.5 M, purity 99.9%) was mixed with 50 mL of DW, and then the admixture was magnetically agitated for 15 min. The medium pH (12) was adjusted by NaOH. Then, the solution was magnetically further stirred for 3 h until a brown precipitate was produced. The precipitate was washed with ethanol and DW, followed by drying to obtain a powder. Finally, the powder was annealed at 450 °C for CuO-NPs production [24].

### **C**haracterization of Al_2_ O_3_ -NPs and CuO-NPs

Al_2_O_3_-NPs and CuO-NPs were subsequently characterized by X-ray diffraction (XRD) patterns, which were recorded from 4 °C to 80 °C using Philips PW 1710 X-ray diffractometer with a Cu Kα as X-ray source. In addition, the images of the produced NPs were analyzed by scanning electron microscopy (SEM, T200 JEOLeol, Japan) to identify the morphology and particle size.

### Establishment of in vitro cultures and application of Al_2_O_3_-NPs and CuO-NPs

We chose laboratory conditions and an in vitro system for this study as key methods to guarantee the distribution of constant concentrations of NPs in the growth media compared to field trials. Seeds were employed as the experimental material for this study. Thereby, fruits were collected from the mature citrus trees of key lime (*Citrus aurantifolia* L.) within the period of December till April. Trees were grown in the Experimental Farm of the Pomology Department, Faculty of Agriculture, Assiut University, Egypt. Seeds were extracted from the fresh fruits and washed under running tap water for 30 min. Healthy seeds were pre-sterilized before germination in a hydrochloric acid solution (30% w/v) for 20 min. After washing several times, seeds were water-saturated at 4 °C for an overnight before culturing. The pretreated uniform seeds were then moved to a laminar flow cabinet and surface-sterilized by immersing them in 30% commercial bleach (sodium hypochlorite < 5.2% (w/v)) for 20 min with continuous shaking. Sterilized seeds were followed by several sterile distilled water changes at least 4–5 times, then they were inoculated in half-strength Murashige and Skoog (MS) medium [25] fortified with 3% (w/v) sucrose and free of plant hormones. For Al_2_O_3_-NPs and CuO-NPs treatments, the same above medium was used and supplemented with suspensions of Al_2_O_3_-NPs or CuO-NPs-based products at three dosages: 100, 200, and 500 mg/L. Sonication for 20 min was done to disperse NPs prior to application. In all media, the pH was stabilized at 5.8 prior to the addition of Gelrite (0.22% w/v) and autoclaving for 20 min at 121 °C. Each culture vessel contained 50 mL of medium and five seeds. Then jars were sustained in a growing room at 25 ± 2 °C in 8 h dark followed by 16 h photoperiod under photosynthetic flux density of 60 µmol m^− 2^ s^− 1^ delivered by cooling white fluorescent tubes. The conditions of the growing room remained constant during the experimental period. Then, the seedlings were harvested for morpho-physiological characterization. A complete randomized design (CRD) was used for this study. Media without NPs were served as control.

### Evaluation of germination frequency and growth indices

After 40 days, we estimated the germination frequency of seeds from various treatments using the following formula:

Germination percentage (%) = $$\:\frac{\mathrm{N}\mathrm{u}\mathrm{m}\mathrm{b}\mathrm{e}\mathrm{r}\:\mathrm{o}\mathrm{f}\:\mathrm{g}\mathrm{e}\mathrm{r}\mathrm{m}\mathrm{i}\mathrm{n}\mathrm{a}\mathrm{t}\mathrm{e}\mathrm{d}\:\mathrm{s}\mathrm{e}\mathrm{e}\mathrm{d}\mathrm{s}}{\mathrm{t}\mathrm{o}\mathrm{t}\mathrm{a}\mathrm{l}\:\mathrm{n}\mathrm{u}\mathrm{m}\mathrm{b}\mathrm{e}\mathrm{r}\:\mathrm{o}\mathrm{f}\:\mathrm{s}\mathrm{e}\mathrm{e}\mathrm{d}\mathrm{s}}\times100$$ 

To quantify changes in growth performance, several classical measures of plant morphology were counted. These including, fresh weight (g), dry weight (g), shoot length (cm), root length (cm), root number, leaf number, and shoot number/explant. For the dry mass assessment of collected tissues, samples were oven-dried at 65 °C till achieving a constant weight.

### Determination of photosynthetic pigments

The concentrations of chlorophyll a (Chl a), chlorophyll b (Chl b), and carotenoids (CAR) in fresh leaves were assessed by dipping chopped leaves in ethanol (95%) overnight as described in the methodology of Lichtenthaler and Wellburn [26]. Then, the optical density (OD) of the extracted solutions was recorded at three wavelengths of 663, 644, and 470 nm for Chl a, Chl b, and CAR, respectively.

### Quantification of reactive oxygen radicals and stress biomarkers

Hydrogen peroxide (H_2_O_2_) was assessed spectrophotometrically at 410 nm by using trichloroacetic acid (TCA) as a reagent and different concentrations of H_2_O_2_ as standards [27]. Superoxide anion (O_2_^•−^) was conducted by monitoring the development of nitrite from hydroxylamine at 530 nm following the methodology of Yang et al. [28]. Hydroxyl radical (^•^OH) was estimated based on the technique of Halliwell et al. [29] via measuring the produced ^•^OH in tissues neutralized in phosphate buffer, including 2-deoxy-D-ribose. Lipid peroxidation was assayed in treated and control samples depending on the thiobarbituric acid reaction by observing malondialdehyde development as reported by Rao and Sresty [30]. Methylglyoxal (MG) was estimated in leaves of lime plants where the dinitrophenylhydrazine-HCl reagent was employed, and the spectrophotometer record was observed after 45 min at 432 nm [31]. Lipoxygenase activity (LOX/EC.1.13.11.1) was analyzed by following the approach of Minguez-mosquera et al. [32] through using potassium phosphate as an extraction buffer (pH 6). LOX activity was observed on a spectrophotometer following the increase in absorbance at 234 nm.

### Analyses of secondary metabolites

Secondary metabolites, including phenolics, flavonoids, anthocyanins, and AsA were analyzed in lime tissues. Phenolics were estimated as pronounced by the scheme of Aery [33], depending on the Folin-Ciocalteu reagent on the methanolic tissues extracts. The spectrophotometer was read at 725 nm, exploiting gallic acid as a blank sample. The quantification of total flavonoids was performed in the methanolic extract of tissues as described by Zou et al. [34], using quercetin as a standard and then reading the samples’ OD at 510 nm. Anthocyanins were quantified in tissues suspended in acidified methanol using a spectrophotometer at 550 nm [35]. AsA was extracted and mixed with TCA using Folin-Ciocalteu reagent, and the OD was noted at the wavelength of 760 nm [36]. Furthermore, the concentration of endogenous α-tocopherol was calculated based on the method of Kivcak and Mert [37] in chloroform extract using 2,2′-dipyridyl and ferric chloride reagents. The developed color was noted at 522 nm spectrophotometrically by using a calibration curve of α-tocopherol.

### Assessment of enzymatic antioxidants

To purify and assess the antioxidant enzyme activities in different samples, a crude enzyme extract was obtained by grinding the leaf tissue in liquid nitrogen before homogenizing in potassium phosphate buffer (pH 7.8), including ethylenediamine tetraacetic acid (EDTA) and polyvinylpyrrolidone (PVP). Thereafter, the mixture was placed in a centrifuge tube and exposed to a cold centrifugal force of 11,500×*g* for 30 min. The relative activities of the determined enzymes were expressed on a protein basis as recommended by Lowry et al. [38].

In reference to the previous assay of Misra and Fridovich [39], superoxide dismutase (SOD/EC.1.15.1.1) activity was analyzed in a mixture consisting of 4 components: sodium carbonate buffer (pH 10.2) + EDTA + epinephrine + enzyme extract. The alteration in OD (in dark) at 480 nm for a minute was checked spectrophotometrically. Catalase (CAT/EC.1.11.1.6) activity was recorded after observing the depletion of H_2_O_2_ for 60 s at 240 nm according to Aebi [40] with slight adjustments supplied by Noctor et al. [41]. Ascorbate peroxidase (APX/EC.1.11.1.11) activity was conducted by detecting the oxidation of ascorbate at 290 nm in a mixture containing H_2_O_2_ and ascorbate [42] through the adjustments assumed by Silva et al. [43]. Glutathione peroxidase (GPX/EC.1.11.1.9) analysis was detected in an extract immersed in a mix of potassium phosphate buffer (pH 7) + reduced glutathione + Na_2_HPO_4_ + 5,5′-dithio-bis-2-nitrobenzoic acid via the methodology of Flohé and Günzle [44], where the OD was verified after 5 min at 412 nm. The assay methods of Habig et al. [45] and Ghelfi et al. [46] were applied to quantify glutathione-S-transferase (GST/EC.2.5.1.18) activity in a mixture of phosphate buffer (pH 6.5), reduced glutathione, and 1-chloro-2,4-dinitrobenzene and then checking the OD value at 340 nm for 3 min. The method of Kumar and Khan [47] was used to determine polyphenol oxidase (PPO/EC.1.10.3.1) activity by monitoring the purpurogallin development at 495 nm. Considering phenylalanine ammonia-lyase (PAL/EC.4.3.1.5) assay, the applied method of Sykłowska-Baranek et al. [48] with minor modifications was followed by incubating the enzyme extract, borate buffer (pH 8.9), and phenylalanine for 1 h at 30ºC, and later HCl (2 M) was mixed thoroughly to block the reaction. Then, the formed trans-cinnamic acid was detected at 290 nm. The alterations in the concentrations of soluble peroxidases (SPO) and ionic peroxidases (IPO) were evaluated in leaf tissue extract based on the established protocol of Ghanati et al. [49], which identified by the rate of change in the absorbance at 470 nm, exploiting guaiacol in phosphate buffer and H_2_O_2_.

### Determination of chelation molecules

The content of reduced glutathione (GSH) in sampled leaves was determined as described by Ellman [50]. Phytochelatins (PCs) were quantified by the subtraction of the total quantity of GSH from non-protein thiols as documented by Nahar et al. [51] through adding the homogenized leaves with sulfosalicylic acid to a reaction mixture of Ellman [50]. Metallothionines (MTCs) were quantified following the detailed study of Cataldo et al. [52]. Nitric oxide (NO) production was monitored using the procedure explained by Adams et al. [53]. The leaves were macerated in glacial acetic acid and then the supernatant was mixed with Griess reagent and kept for 30 min at room temperature. The absorbance reading was taken at 560 nm. As reported by Nashef et al. [54], hydrogen sulfide (H_2_S) was measured in frozen tissues grounded in K-phosphate buffer and EDTA, then added to 5,5′-dithiobis (2-nitrobenzoic acid), where the OD was recorded at 415 nm.

### Ionomics analysis

The various elements were estimated in seedlings’ dry tissues and expressed as dry weight% using the energy dispersive X-ray analysis (EDX) method (JEOL JSM 5400 LN, England).

### Statistical analysis

The experiment was repeated twice, with three replicates per treatment (6 jars for morphological analysis and 5 jars for physiological analysis for each replicate). Data were statistically analyzed using SPSS (version 25.0). Significant differences in plant traits across different treatments were tested by a Duncan’s multiple comparison test (*P < 0.05)*. The principal component (PCA) and heatmap analyses were executed exploiting software applied on http://www.bioinformatics.com.cn/cgi-bin/guide.cgi.

## Results

### The characterization of Al_2_O_3_-NPs and CuO-NPs

**Fig. 1:**
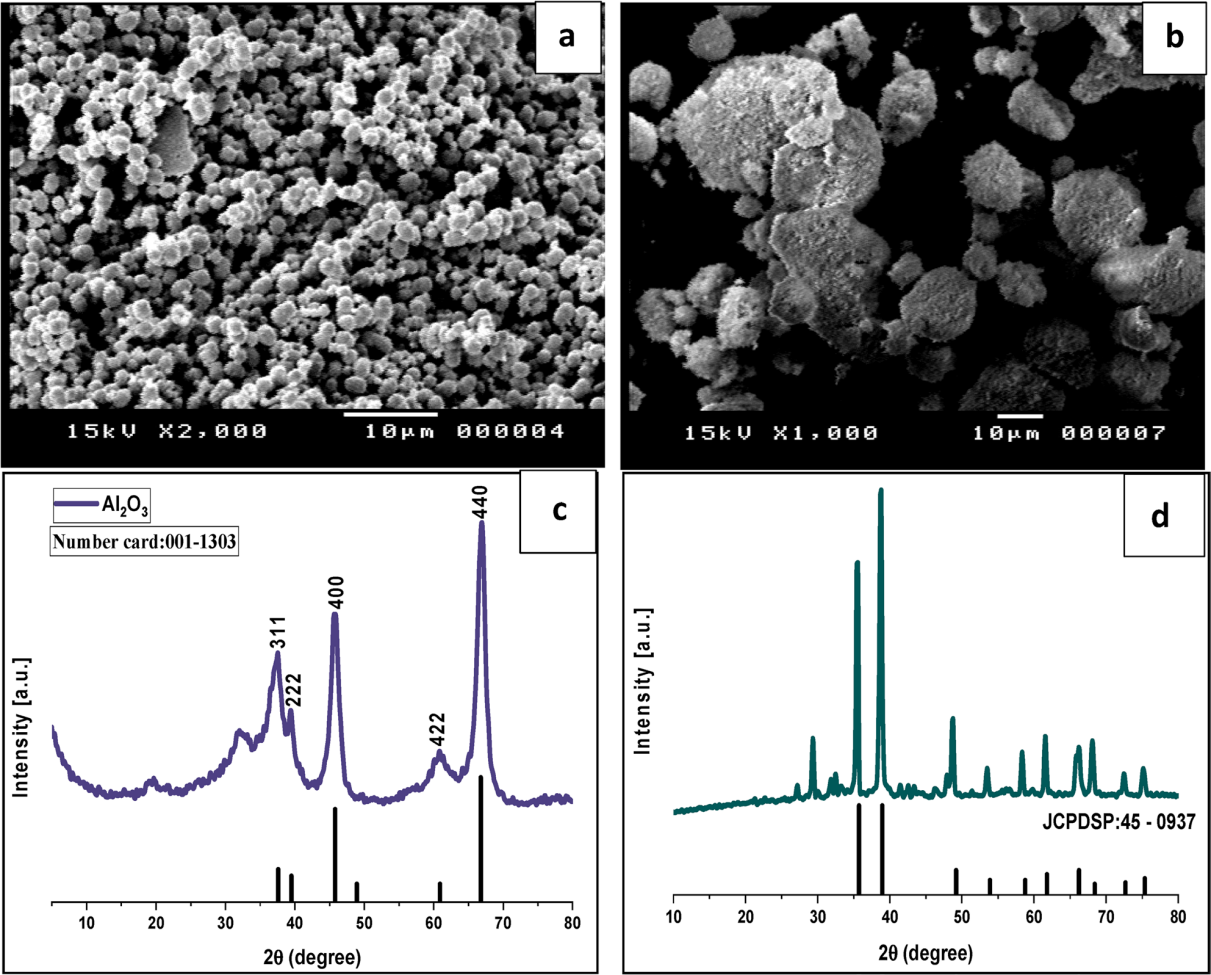
Characterization of nanoparticles: (**a**): SEM image of Al_2_O_3_-NPs, (**b**): SEM images of CuO-NPs, (**c**): XRD of Al_2_O_3_-NPs, and (**d**): XRD of CuO-NPs

As displayed in Fig. 1 (a, b), a representative image of Al_2_O_3_-NPs at solid state, attained by SEM, exhibited the formation of spherical-shaped Al_2_O_3_-NPs with an average size of about 4 nm, and those of CuO-NPs were in a quasi-spherical shape with a size of about 16 nm. The XRD scans of Al_2_O_3_-NPs and CuO-NPs samples confirmed the nanoscale of the prepared nanomaterials and the diffraction peaks revealed notable homogeneity with high purity (Fig. 1c, d).

### The influence of Al_2_O_3_-NPs and CuO-NPs on germination and phenotyping

**Fig. 2:**
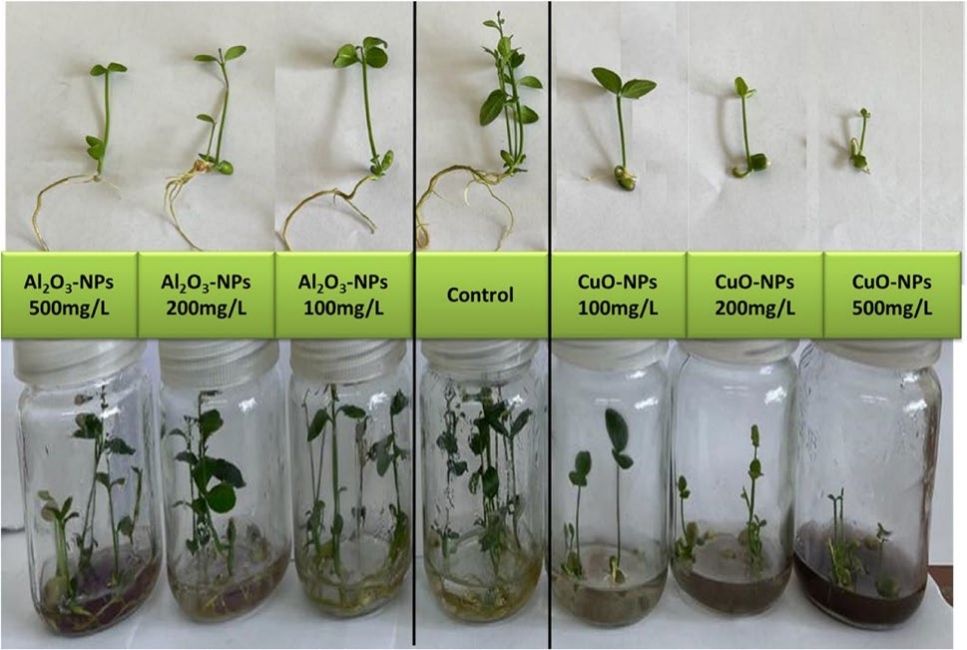
Phenotypic responses of lime (*Citrus aurantifolia*) seedlings grown in vitro under different concentrations of Al_2_O_3_-NPs and CuO-NPs

The application of both Al_2_O_3_-NPs and CuO-NPs had diverse impacts on the germination frequency and growth features, as shown in Table 1. Al_2_O_3_-NPs at 100 mg/L did not significantly affect the germination rate, however, higher concentrations of 200 and 500 mg/L considerably reduced germination capacity by 18.7% and 24.2%, respectively, compared to the control. CuO-NPs had a great toxic impact, remarkably reducing germination values by 60%, 50.5%, and 49.5%, at concentrations of 100, 200, and 500 mg/L, respectively. Additionally, the use of Al_2_O_3_-NPs revealed a non-significant effect on the morphological characteristics of lime seedlings, except for a significant reduction in the dry weight, root length, and root number, mostly at 500 mg/L. Conversely, CuO-NPs-treated plants exhibited a significant decrease in all morphological variables even at the lowest dosage (100 mg/L), except for shoot number. The most pronounced reductions in fresh weight (55%), dry weight (82.4%), shoot length (74.5%), shoot number (19.6%), root length (96.7%), root number (56%), and leaf number (44.3%) were noted at 500 mg/L CuO-NPs relative to the control values. The visible influence of NPs included deficient growth, shorter shoots and roots, and chlorosis of leaves, especially for CuO-NPs-treated plants (Fig. 2).

**Table 1:** The germination percentage and growth parameters of lime (*Citrus aurantifolia*) seedlings grown in vitro under different treatments of Al_2_O_3_-NPs and CuO-NPs. Variants bearing the different letters are statistically significant at *P* < 0.05

Treatment		Germination percentage (%)	Fresh weight (g)	Dry weight (g)	Shoot length (cm)	Shoot number	Root length (cm)	Root number	Leaf number
	**Ctrl**	91 ± 1.1d	0.20 ± 0.01b	0.17 ± 0.01f	4.31 ± 0.01 cd	1.24 ± 0.07ab	6.33 ± 0.08d	3.23 ± 0.12d	5.08 ± 0.32bc
Al_2_O_3_-NPs (mg/L)	**100** **200**	85 ± 2.9 cd 74 ± 1.7bc	0.20 ± 0.020b 0.19 ± 0.030b	0.15 ± 0.007ef 0.13 ± 0.010e	4.23 ± 0.14 cd 4.41 ± 0.11 cd	1.26 ± 0.14ab 1.41 ± 0.02b	4.87 ± 0.27c 5.69 ± 0.01 cd	2.88 ± 0.22d 2.92 ± 0.04d	6.54 ± 0.87c 6.08 ± 0.31bc
	**500**	69 ± 1.8b	0.17 ± 0.020b	0.12 ± 0.004de	3.72 ± 0.05c	1.11 ± 0.05ab	4.92 ± 0.22c	2.00 ± 0.22c	5.45 ± 0.12bc
CuO-NPs (mg/L)	**100**	36 ± 3.1a	0.11 ± 0.010a	0.08 ± 0.010c	2.11 ± 0.27b	1.42 ± 0.08b	0.55 ± 0.23b	1.13 ± 0.26ab	4.32 ± 0.23b
	**200**	45 ± 2.90a	0.10 ± 0.004a	0.06 ± 0.011b	1.50 ± 0.13ab	1.08 ± 0.08ab	0.27 ± 0.08a	1.17 ± 0.08ab	4.33 ± 0.22b
	**500**	46 ± 2.5a	0.09 ± 0.002a	0.03 ± 0.001a	1.10 ± 0.08a	1.00 ± 0.008a	0.21 ± 0.11a	1.42 ± 0.17a	2.83 ± 0.22a

Variants bearing the different letters are statistically significant at *P* <0.05

### The impact of Al_2_O_3_-NPs and CuO-NPs on photosynthetic pigments

**Fig. 3:**
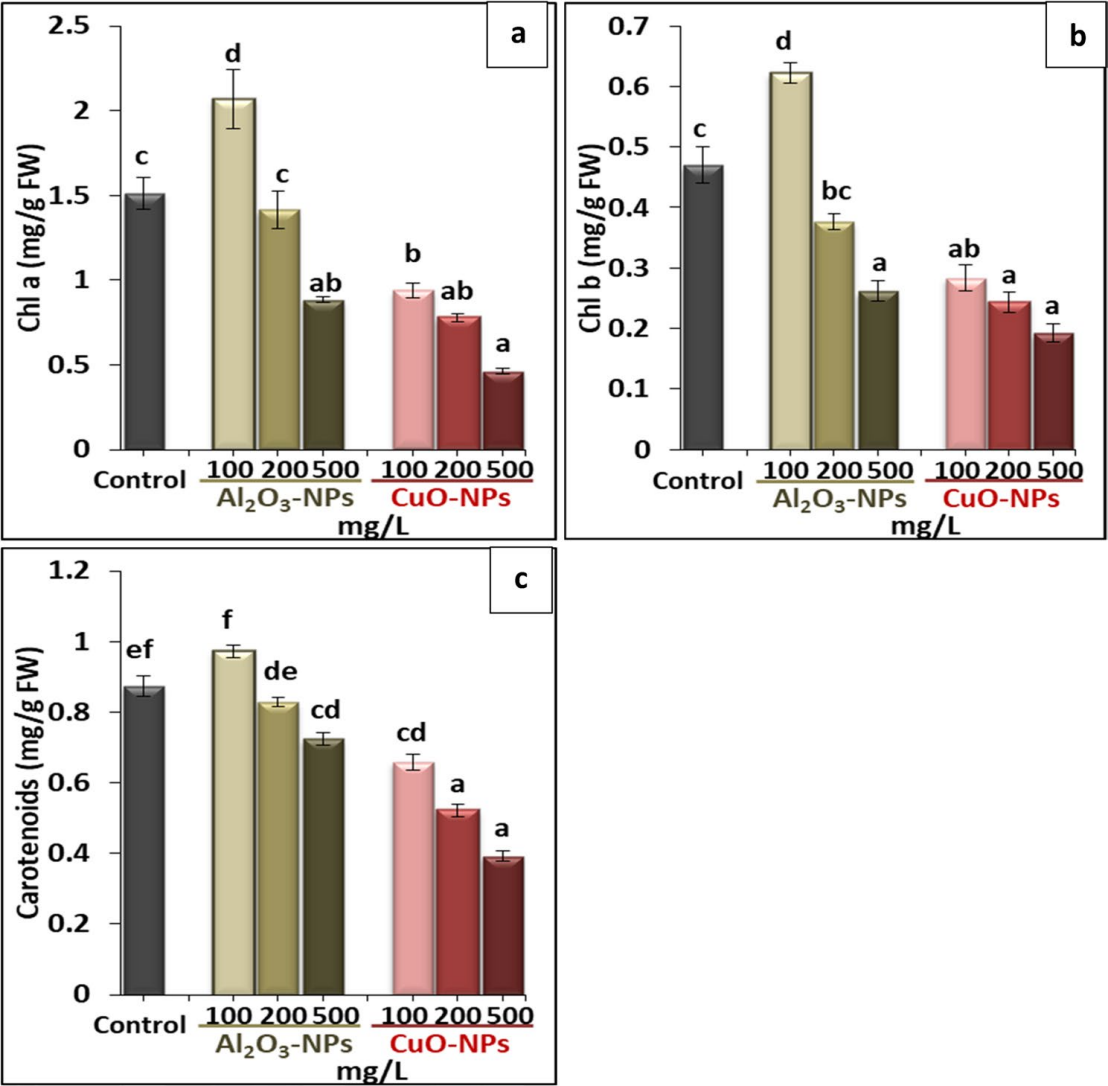
Effect of different concentrations of Al_2_O_3_-NPs and CuO-NPs on photosynthetic pigments of lime seedlings; **a**: chlorophyll a Chl a, (**b**): chlorophyll b Chl b, and (**c**): carotenoids. Different letters reveal significant differences among the treatments at *p* ˂ 0.05, based on Duncan’s multiple range test

The data of photosynthetic pigments are illustrated in Fig. 3 (a−c). The lowest Al_2_O_3_-NPs concentration (100 mg/L) was the only treatment that boosted the biosynthesis of Chl a, Chl b, and CAR by 37.1%, 31.9%, and 11.5%, respectively, in respect to the control. While all other concentrations of Al_2_O_3_-NPs and CuO-NPs had negative impacts on Chl a, Chl b, and CAR, showing a clear decrease by increasing NPs dose in the media. Meanwhile, plants showed higher sensitivity to CuO-NPs than Al_2_O_3_-NPs, with greatest reductions of 69.5%, 59.6%, and 55.2% for Chl a, Chl b, and CAR, respectively, at 500 mg/L CuO-NPs, in comparison with the control. The change in leaf color from dark green to light green was observed in NPs-stressed plants, which may reflect the biodegradation of green chlorophyll pigment. This disorder was more pronounced under CuO-NPs treatments, as shown in Fig. 2.

### The impact of Al_2_O_3_-NPs and CuO-NPs on oxidative stress markers

**Fig. 4:**
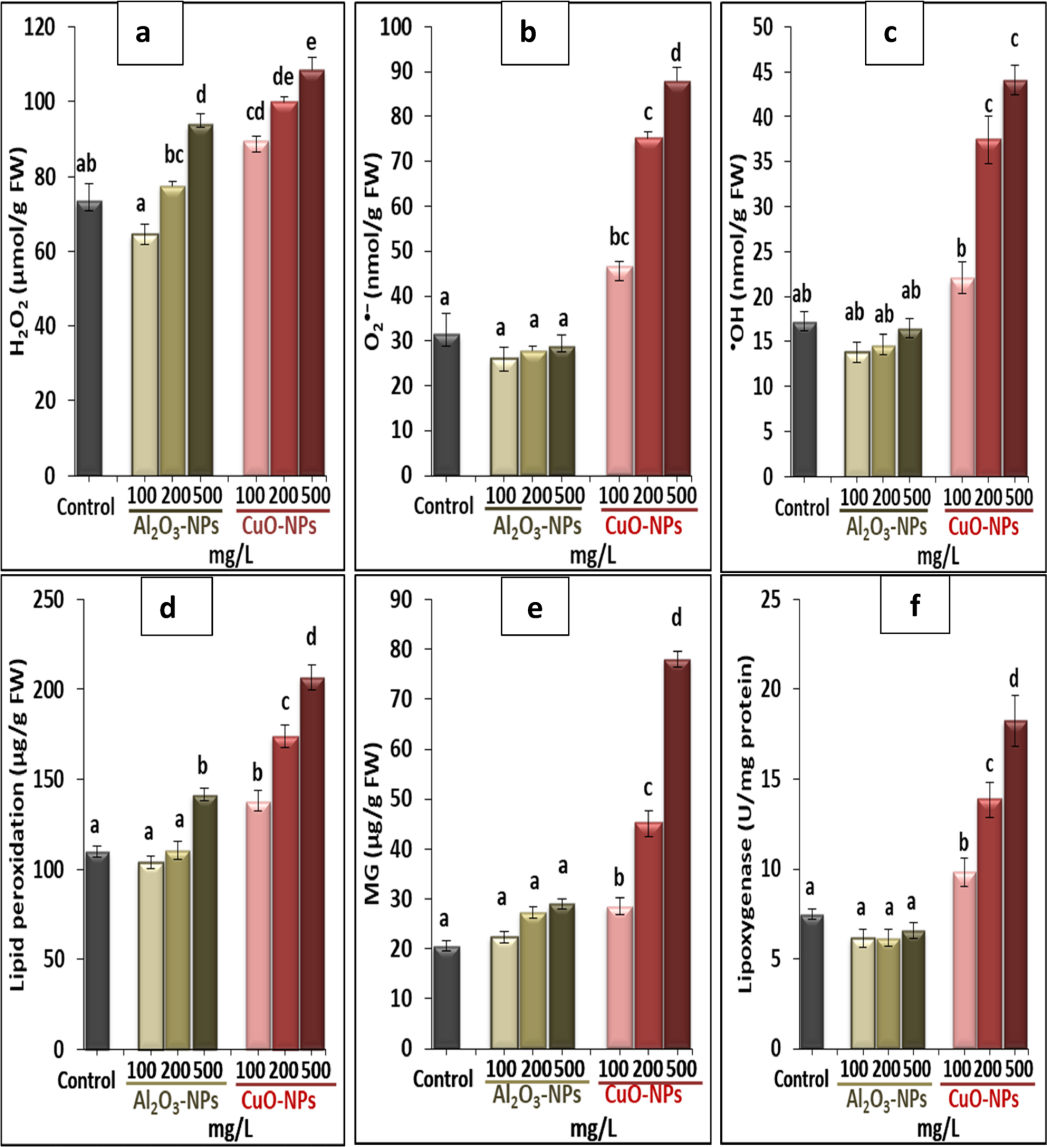
Effect of different concentrations of Al_2_O_3_-NPs and CuO-NPs on (**a**): hydrogen peroxide H_2_O_2_, (**b**): superoxide anion O_2_^•−^, (**c**): hydroxyl radical ^•^OH, (**d**): lipid peroxidation, (**e**): methylglyoxal MG, and (**f**): lipoxygenase activity LOX in lime seedlings. Different letters reveal significant differences among the treatments at *p* ˂ 0.05, based on Duncan’s multiple range test

The oxidative stress markers (H_2_O_2_, O_2_^•−^, ^•^OH, lipid peroxidation, MG, and LOX) were analyzed to evaluate the level of oxidative stress triggered by Al_2_O_3_-NPs and CuO-NPs. As shown in Fig. 4 (a−f), both Al_2_O_3_-NPs and CuO-NPs affected the stimulation of oxidative stress biomarkers in a dose-and species-dependent manner. Notably, 500 mg/L Al_2_O_3_-NPs raised the levels of only H_2_O_2_ and lipid peroxidation by 1.3- and 1.3-fold, respectively, compared to the control, but did not affect other oxidative markers. On the contrary, CuO-NPs at different concentrations markedly elevated all stress markers in response to the increased concentration of CuO-NPs in the media. The greatest peak of CuO-NPs was denoted at the level of 500 mg/L, showing 1.5-, 2.8-, 2.6-, 2.45-, 3.8-, and 1.9-fold increases in H_2_O_2_, O_2_^•−^, ^•^OH, lipid peroxidation, MG, and LOX, respectively, over the control, indicating the severe toxicity induced by CuO-NPs compared to Al_2_O_3_-NPs, especially at higher levels.

### The impact of Al_2_O_3_-NPs and CuO-NPs on secondary metabolites

**Fig. 5:**
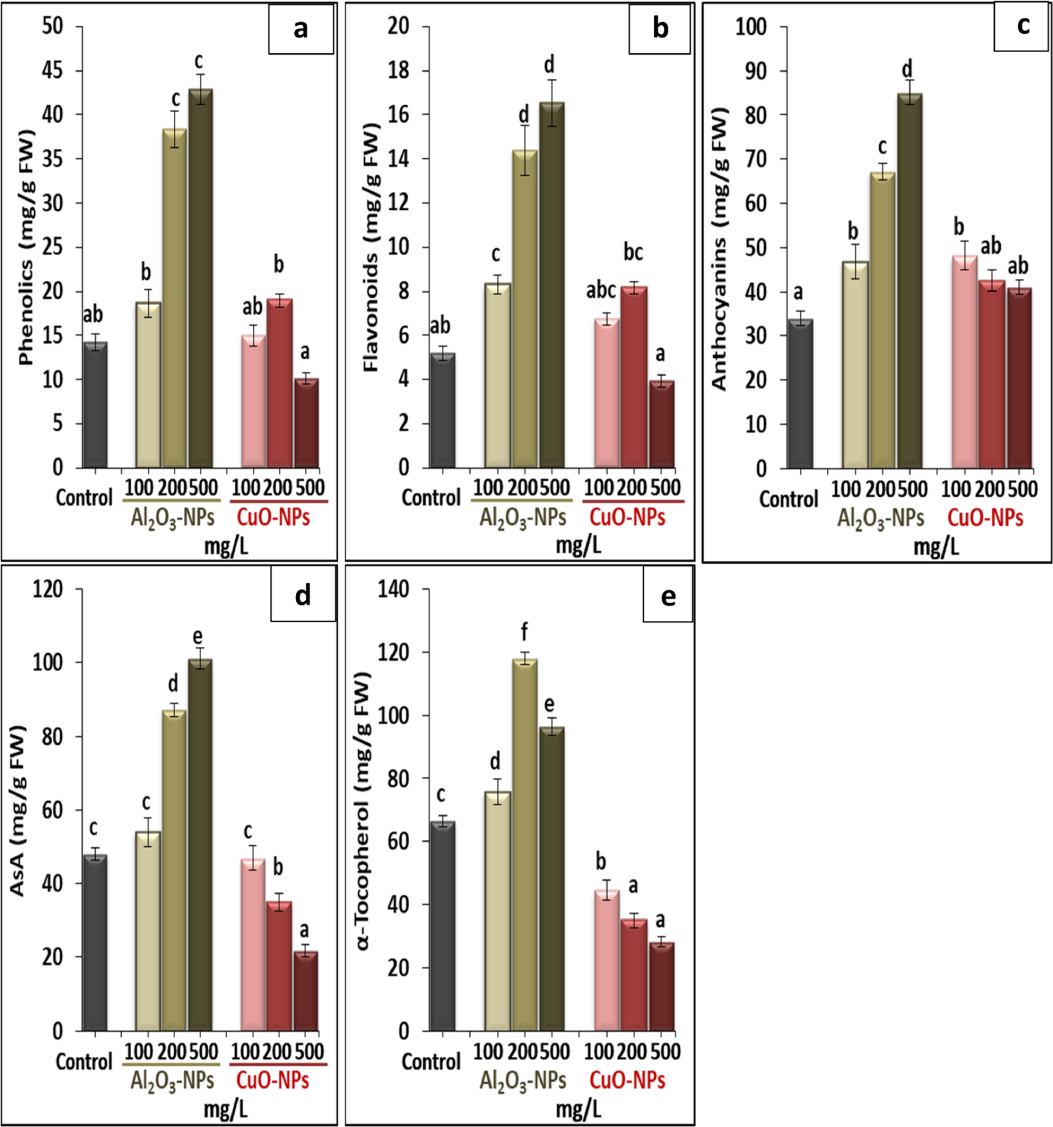
Effect of different concentrations of Al_2_O_3_-NPs and CuO-NPs on (**a**): phenolics, (**b**): flavonoids, (**c**): (**d**): anthocyanins, and (**e**): α-tocopherol in lime seedlings. Different letters reveal significant differences among the treatments at *p* ˂ 0.05, based on Duncan’s multiple range test

The results in Fig. 5 (a−e) revealed that the application of Al_2_O_3_-NPs to the growing medium had positive effects on the contents of phenolics, flavonoids, anthocyanins, AsA, and α-tocopherol compared to the control. According to the results, Al_2_O_3_-NPs (500 mg/L) resulted in the highest production of phenolics, flavonoids, anthocyanins, and AsA by 201.3%, 219.3%, 151%, and 110.6%, respectively, over their control. However, α-tocopherol was obviously increased by 77.5% under the treatment of 200 mg/L Al_2_O_3_-NPs. On the other hand, when plants were subjected to CuO-NPs, they showed non-significant changes in the levels of phenolics, flavonoids, and anthocyanins, while the exposure to CuO-NPs decreased the levels of AsA and α-tocopherol. The treatment of 500 mg/L CuO-NPs induced the highest reduction in the contents of AsA and α-tocopherol by 54.8% and 57.6%, respectively, against the control.

### The impact of Al_2_O_3_-NPs and CuO-NPs on enzymatic antioxidants

**Fig. 6:**
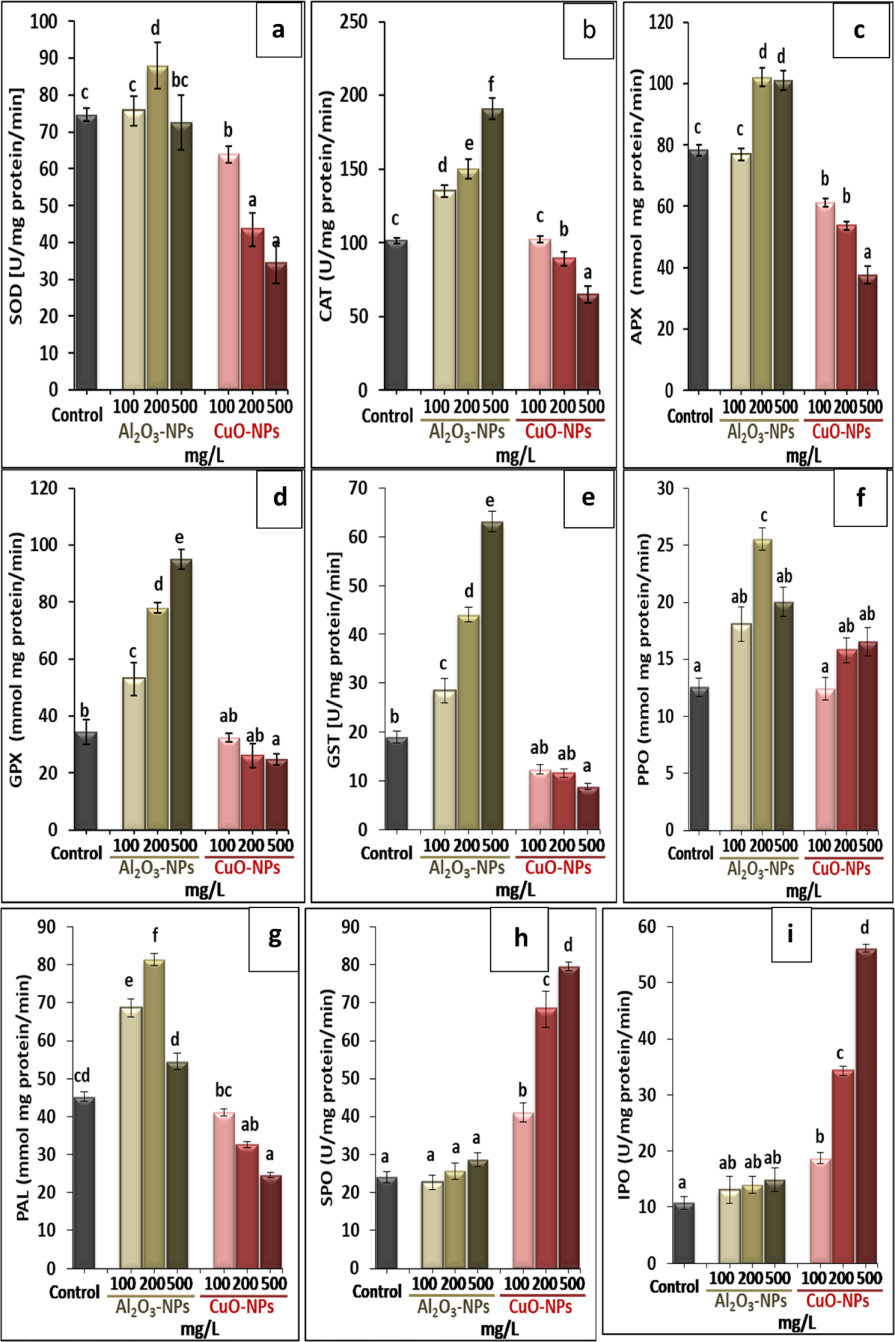
Effect of different concentrations of Al_2_O_3_-NPs and CuO-NPs on (**a**): superoxide dismutase SOD, (**b**): catalase CAT, (**c**): ascorbate peroxidase APX, (**d**): glutathione peroxidase GPX, (**e**): glutathione-*S*-transferase GST, (**f**): polyphenol oxidase PPO, (**g**): phenylalanine ammonia-lyase PAL, (**h**): soluble peroxidase SPO, and (**i**): ionic peroxidase IPO in lime seedlings. Different letters reveal significant differences among the treatments at p ˂ 0.05, based on Duncan’s multiple range test

The inclusion of Al_2_O_3_-NPs in the plant medium enhanced the activities of antioxidant enzymes compared with the untreated control. At 200 mg/L Al_2_O_3_-NPs, the activities of SOD, APX, PPO, and PAL displayed the most significant increases by 17.8%, 30.2%, 103.6%, and 79.8%, respectively, whereas 500 mg/L Al_2_O_3_-NPs greatly boosted the activities of CAT, GPX, and GST by 88.3%, 175.4%, and 233.8%, respectively, versus the control. The activities of SPO and IPO did not significantly change whatever the added level of Al_2_O_3_-NPs. In a different trend, the exposure of lime plants to CuO-NPs led to a decline in the activities of SOD, CAT, APX, GPX, and PAL compared with the untreated plants. The maximum decreases by 53.8%, 35.9%, 51.9%, 28.3%, and 45.6% were recorded at the level of 500 mg/L CuO-NPs for the above-mentioned enzymes, respectively. Moreover, CuO-NPs strongly promoted the activities of SPO and IPO in lime tissues and the best elevation in their activities by 231.1% and 422%, respectively, was noted at 500 mg/L CuO-NPs (Fig. 6a−i).

### The effect of Al_2_O_3_-NPs and CuO-NPs on chelation molecules, NO, and H_2_S contents

**Fig. 7:**
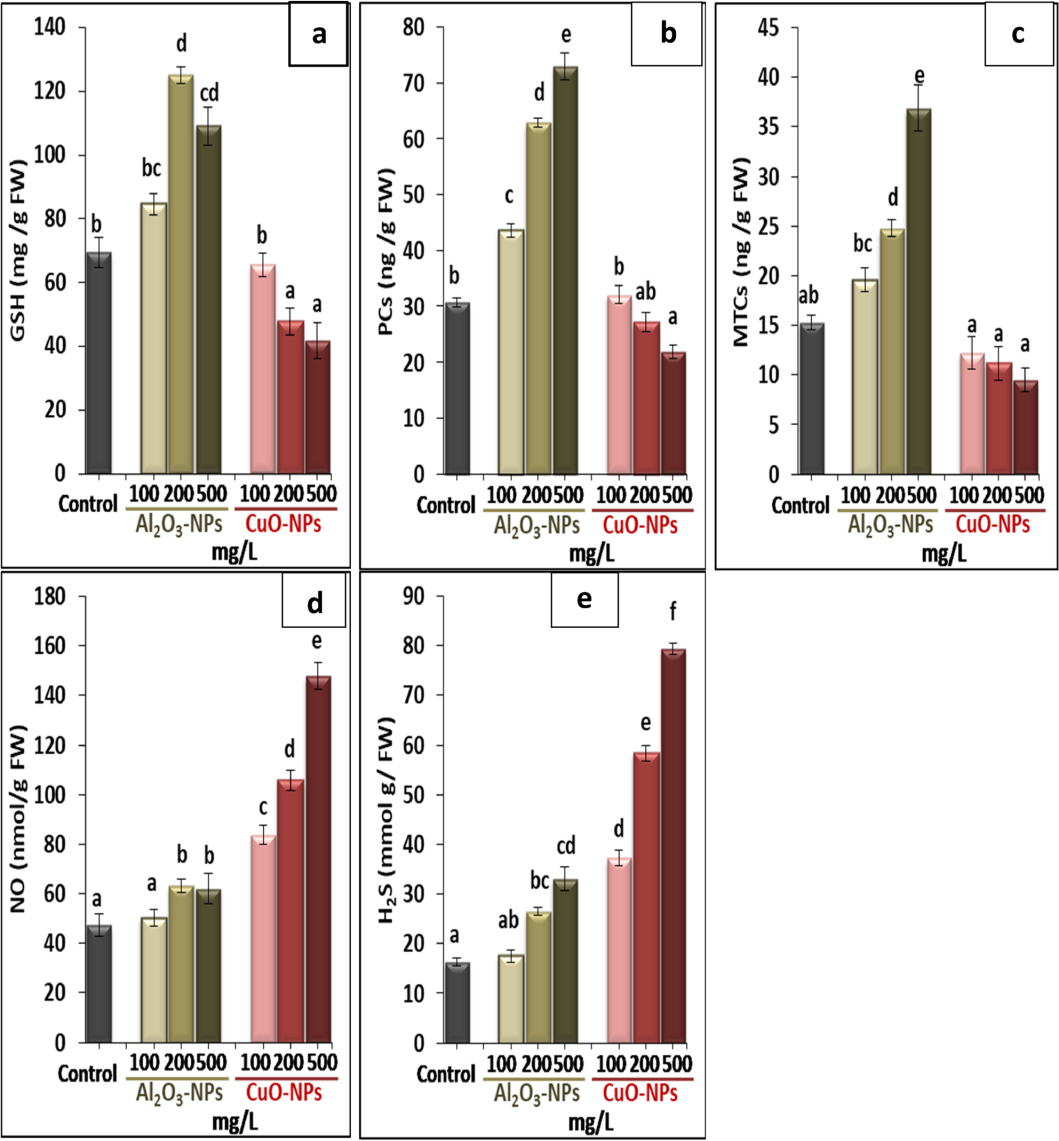
Effect of different concentrations of Al_2_O_3_-NPs and CuO-NPs on (**a**): reduced glutathione GSH, (**b**): phytochelatins PCs, (**c**): metallothionines MTCs, (**d**): nitric oxide NO, and (**e**): hydrogen sulfide H_2_S in lime seedlings. Different letters reveal significant differences among the treatments at p ˂ 0.05, based on Duncan’s multiple range test

The contents of endogenous chelators, i.e., GSH, PCs, and MTCs, in response to Al_2_O_3_-NPs and CuO-NPs treatments are summarized in Fig. 7 (a−f). The plants cultured in media amended with Al_2_O_3_-NPs evoked higher amounts of chelation molecules compared with those cultured on free-NPs media. The highest content of GSH (125.09 mg/g FW) was noticed at 200 mg/L Al_2_O_3_-NPs, while the maximum enhancements of PCs and MTCs (72.94 and 36.86 ng/g FW, respectively) were denoted at 500 mg/L Al_2_O_3_-NPs. On the other hand, the diverse concentrations of CuO-NPs negatively impacted the contents of GSH, PCs, and MTCs in linear correlation to record the minimum values of 41.73 mg/g FW, 9.53 ng/g FW, and 21.88 ng/g FW, respectively, at 500 mg/L CuO-NPs. The contents of NO and H_2_S in the plants were remarkably improved by both types of NPs, and the most increases were noticed by CuO-NPs treatments, particularly at 500 mg/L, showing an increase of 212.1% and 386.9%, respectively, compared with the control.

### The influence of Al_2_O_3_-NPs and CuO-NPs on ion content

Data in Table 2 indicate that aluminum (Al) content in the plant tissues was increased with increasing Al_2_O_3_-NPs level in the media, reaching a maximum of 1.9 times higher than the control at 500 mg/L Al_2_O_3_-NPs. However, at the same concentration, all other nutrient elements were decreased except for a substantial rise in the contents of Si, P, Ca, and Fe. In CuO-NPs-treated plants, the content of Cu remarkably increased in a linear relation with elevating CuO-NPs in the growing medium, achieving the highest accumulation level (15.6-fold) relative to the control at 500 mg/L CuO-NPs. Variation in element accumulation was recorded at the different dosages of CuO-NPs, but at the highest level of CuO-NPs (500 mg/L), only S and Ca displayed a marked increase compared with the control, while other elements showed varied levels of decrease.

**Table 2:** Various nutrients of lime (*Citrus aurantifolia*) seedlings grown in vitro under different treatments of Al_2_O_3_-NPs and CuO-NPs. Variants bearing the different letters are statistically significant at *P* <0.05

Element	Control	Al_2_O_3_-NPs (mg/L)			CuO-NPs (mg/L)		
		100	200	500	100	200	500
Mg Al Si P S Cl K Ca Fe Cu Zn	08.01 ± 1.05d 33.85 ± 4.09c 10.49 ± 1.35b 2.37 ± 0.35c 0.65 ± 0.03b 5.01 ± 0.97e 1.23 ± 0.17e 09.87 ± 1.23e 0.30 ± 0.02b 2.98 ± 0.75b 2.59 ± 0.059e	22.04 ± 3.21f 36.10 ± 3.21c 21.49 ± 2.89d 7.26 ± 1.02e 0.00 ± 0.00a 13.93 ± 2.3f 2.23 ± 0.53f 0.61 ± 0.23a 5.19 ± 1.01e 0.92 ± 0.09a 0.22 ± 0.05ab	3.81 ± 0.53b 40.87 ± 5.1d 38.59 ± 3.29e 2.76 ± 0.65c 0.00 ± 0.0a 1.28 ± 0.21b 0.71 ± 0.09d 6.77 ± 0.09d 2.46 ± 0.64d 4.66 ± 0.78c 1.91 ± 0.28d	0.00 ± 0.0a 64.64 ± 6.3e 17.75 ± 2.1c 4.45 ± 0.74d 0.81 ± 0.04b 3.81 ± 0.65d 0.31 ± 0.06bc 1.33 ± 0.79b 0.62 ± 0.09c 2.00 ± 0.82b 0.45 ± 0.08b	10.86 ± 0.85e 3.41 ± 0.21a 3.48 ± 0.31a 0.28 ± 0.02b 2.76 ± 0.38c 2.15 ± 0.43bc 0.17 ± 0.02a 3.25 ± 0.34c 0.01 ± 0.00a 16.42 ± 1.78d 0.16 ± 0.01a	6.32 ± 0.61c 3.99 ± 0.42a 3.76 ± 0.19a 0.00 ± 0.00a 4.26 ± 0.79d 5.24 ± 0.88d 0.34 ± 0.05bc 3.12 ± 0.29c 0.45 ± 0.08bc 30.72 ± 4.09e 0.62 ± 0.02c	5.16 ± 0.43c 5.03 ± 0.6b 3.29 ± 0.22a 0.00 ± 0.0a 4.81 ± 0.86d 0.48 ± 0.01a 0.15 ± 0.03a 1.04 ± 0.09b 0.31 ± 0.07b 46.53 ± 3.2f 2.20 ± 0.47e

Variants bearing the different letters are statistically significant at *P* <0.05

Abbreviations: *Mg * magnesium, *Al* aluminum, *Si* silicon, *P* phosphor, *S* sulfur, *Cl* chloride, *K* potassium, *Ca* calcium, *Fe* iron, *Cu* copper, and *Zn* zinc

### The correlation analysis of the data collected using PCA and heatmap analysis

**Fig. 8:**
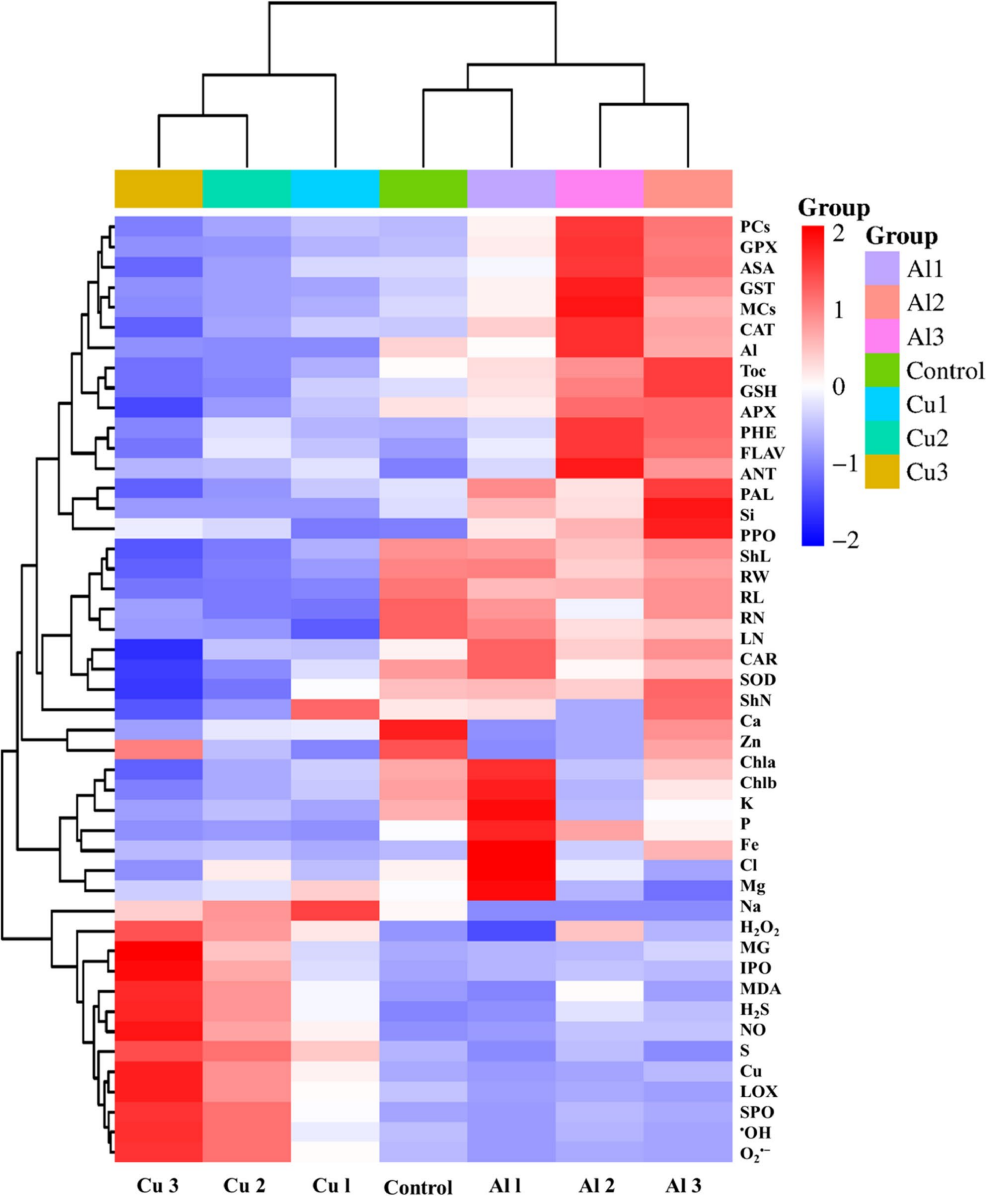
The heatmap analysis of the morpho-physiological studied traits in lime seedlings under different concentrations of Al_2_O_3_-NPs and CuO-NPs. Appreviations; Al1 = Al_2_O_3_-NPs (100 mg/L), Al2 = Al_2_O_3_-NPs (200 mg/L), Al3 = Al_2_O_3_-NPs (500 mg/L), Cu1 = CuO-NPs (100 mg/L), Cu2 = CuO-NPs (200 mg/L), Cu3 = CuO-NPs (500 mg/L), PCs = phytochelatins, GPX = glutathione peroxidase, ASA = ascorbic acid, GST = glutathione-*S*-transferase, MCs = metallothionines, CAT = catalse, Al = Aluminum, Toc = α-tocopherol, GSH = reduced glutathione, APX = ascorbate peroxidase, PHE = phenolics, FLAV = flavonoids, ANT = antioxidants, PAL = phenylalanine ammonia-lyase, S = sulfur, PPO = polyphenol oxidase, ShL = shoot length, RW = fresh weight, RL = root length, RN = root number, LN = leaf number, CAR = carotenoids, SOD = superoxide dismutase, shN = shoot number, Ca = calcium, Zn = zinc, Chla = Chlorophyll a, Chlb = Chlorophyll b, K = phosphate, P = phosphour, Fe = iron, Cl = chloride, Mg = magenesium, Na = sodium, H_2_O_2_ = hydrogen peroxide, MG = methylglyoxal, IPO = ionic peroxidase, MDA = malondialdehyde, H_2_S = hydrogen sulfide, NO = nitric oxide, Si = silicon, Cu = copper, LOX = lipoxygenase activity, SPO = soluble peroxidase, OH = hydroxyl radical, and O_2_ = superoxide anion

Observations on the germination percentage, morpho-physiological traits, and ionomics profile of lime plants under different levels of Al_2_O_3_-NPs and CuO-NPs were used for PCA and heatmap analyses. In Fig. 8, the toxic effect of intensified levels of CuO-NPs was positively correlated to the accumulation of Cu, ROS/RNS (H_2_O_2_, O_2_^•−^, ^•^OH, and NO), and oxidative biomarkers (MG, LOX, and MDA). These parameters negatively affected the pigmentation (Chl a, Chl b, and CAR), chelation agents (GSH, PCs, GST, and MTCs), non-enzymatic molecules, secondary metabolites (PHE, Anth, FlAV, AsA, and Toc), antioxidant enzymes (CAT, SOD, APX, and GPX), and growth parameters (FW, ShN, RN, SL, and RL) as well as minerals (Ca, K, Mg, P, and Si). However, Al_2_O_3_-NPs induced accumulation of antioxidants and chelation agents with mild adverse effects on growth parameters, oxidative stress, and pigmentation.

**Fig. 9:**
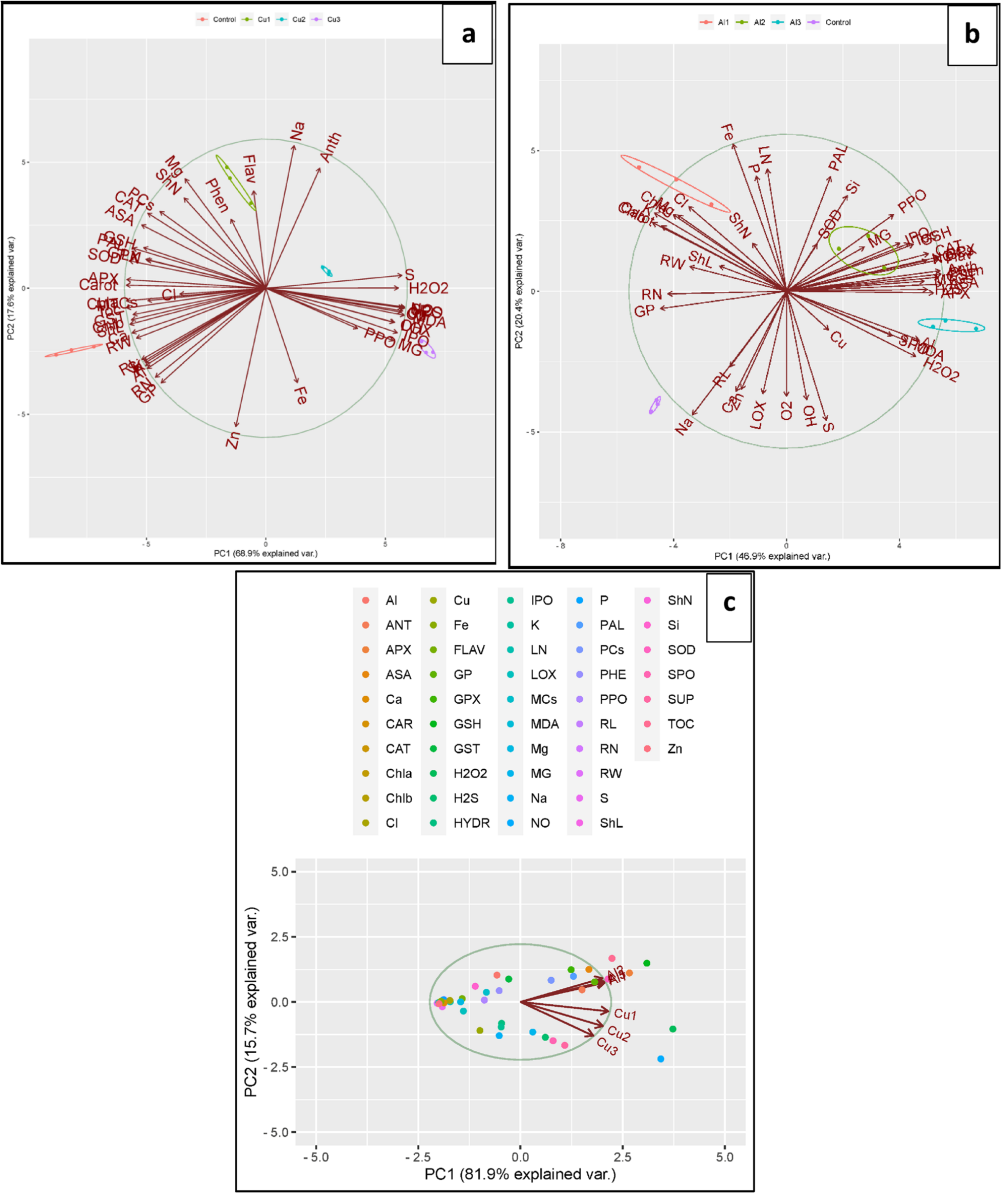
The principal component analysis of the morpho-physiologically studied traits in lime seedlings under the different concentrations of (**a**): Al_2_O_3_-NPs, (**b**): CuO-NPs, and (**c**): the relation of various traits to the applied nanomaterials. Appreviations; Al1 = Al_2_O_3_-NPs (100 mg/L), Al2 = Al_2_O_3_-NPs (200 mg/L), Al3 = Al_2_O_3_-NPs (500 mg/L), Cu1 = CuO-NPs (100 mg/L), Cu2 = CuO-NPs (200 mg/L), Cu3 = CuO-NPs (500 mg/L), Al = Aluminum, ANT = antioxidants, APX = ascorbate peroxidase, ASA = ascorbic acid, Ca = calcium, CAR = carotenoids, CAT = catalse, Chla = Chlorophyll a, Chlb = Chlorophyll b, Cl = chloride, Cu = copper, Fe = iron, FLAV = flavonids, GP = germination percentage, GPX = glutathione peroxidase, GSH = reduced glutathione, GST = glutathione-*S*-transferase, H_2_O_2_ = hydrogen peroxide, H_2_S = hydrogen sulfide, HYDR = hydroxyl radical, IPO = ionic peroxidase, K = phosphate, LN = leaf number, LOX = lipoxygenase activity, MCs = metallothionines, MDA = malondialdehyde, MG = methylglyoxal, Na = sodium, NO = nitric oxide, P = phosphour, PAL = phenylalanine ammonia-lyase, PCs = phytochelatins, PHE = phenolics, PPO = polyphenol oxidase, RL = root length, RN = root number, RW = fresh weight, S = sulfur, ShL = shoot length, shN = shoot number, Si = silicon, SOD = superoxide dismutase, SPO = soluble peroxidase, SUP = superoxide anion, TOC = α-tocopherol, and Zn = zinc

**Fig. 10:**
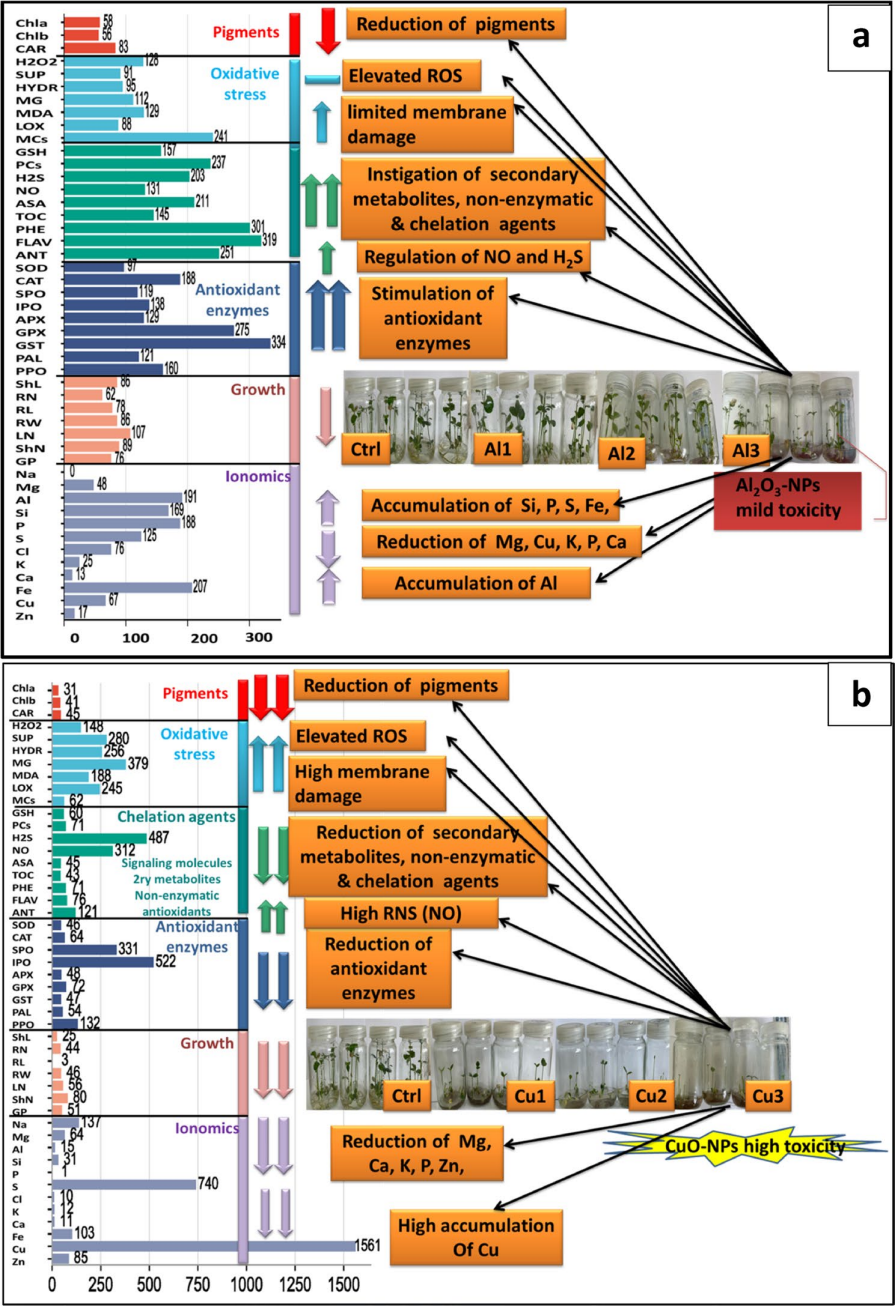
The percent change of the studied traits relative to control plants under (**a**): Al_2_O_3_-NPs and (**b**): CuO-NPs. Appreviations; Al1 = Al_2_O_3_-NPs (100 mg/L), Al2 = Al_2_O_3_-NPs (200 mg/L), Al3 = Al_2_O_3_-NPs (500 mg/L), Cu1 = CuO-NPs (100 mg/L), Cu2 = CuO-NPs (200 mg/L), Cu3 = CuO-NPs (500 mg/L), Chla = Chlorophyll a, Chlb = Chlorophyll b, CAR = carotenoids, H_2_O_2_ = hydrogen peroxide, SUP = superoxide anion, HYDR = hydroxyl radical, MG = methylglyoxal, MDA = malondialdehyde, LOX, MCs = metallothionines, GSH = reduced glutathione, PCs = phytochelatins, H_2_S = hydrogen sulfide, NO = nitric oxide, ASA = ascorbic acid, TOC = α-tocopherol, PHE = phenolics, FLAV = flavonids, ANT = antioxidants, SOD = superoxide dismutase, CAT = catalse, SPO = soluble peroxidase, IPO = ionic peroxidase, APX = ascorbate peroxidase, GPX = glutathione peroxidase, GST = glutathione-*S*-transferase, PAL = phenylalanine ammonia-lyase, PPO = polyphenol oxidase, ShL = shoot length, RN = root number, RL = root length, RW = fresh weight, LN = leaf number, shN = shoot number, GP = germination percentage, Na = sodium, Mg = magenesium, Al = Aluminum, Si = silicon, P = phosphour, S = sulfur, Cl = chloride, K = phosphate, Ca = calcium, Fe = iron, Cu = copper, and Zn = zinc

The PCA analysis presented in Fig. 9B and C demonstrate that the biplot represent the first (PC1) and second (PC2) axes of PCA by 69.9% and 17.6% of the used traits under the impact of various dosages of CuO-NPs, where P1 differentiated the control and Cu1 treatments, whereas PC2 represented Cu2 and Cu3. These features clearly demonstrate the toxic effects of ROS production and Cu accumulation at the level of 500 mg/L. Comparatively, fewer variables representing 46.9% and 20.4% for the PC1 and PC2 components were corresponded to the control and Al1 as well as Al2 and Al3 treatments, respectively. The PCA analysis of Fig. 9C demonstrate the presence of variable responses of lime to different NPs, where Al_2_O_3_-NPs exhibited mild effects and CuO-NPs induced severe responses, with 96.6% of the total variance represented among the analyzed traits. Moreover, the collective traits’ differences based on their percent increase or decrease under Al_2_O_3_-NPs and CuO-NPs treatments relative to the control plants are displayed in Fig. 10.

## Discussion

The current investigation demonstrated that Al_2_O_3_-NPs and CuO-NPs differently affected the seed germination and growth traits of lime; however, CuO-NPs showed stronger toxic effects than Al_2_O_3_-NPs at each exposed concentration. Wu et al. [55] suggested that the absorption of metallic NPs through the seed surface may motivate the release of toxic ions from NPs, which triggers adverse effects on seed germination and tangible growth. The data revealed that Al_2_O_3_-NPs at 100 and 200 mg/L had only minor effects on the germination and growth parameters; nevertheless, 500 mg/L was more toxic to lime growth. In parallel, Akdemir [56] reported that the in vitro germination of barley was not affected by Al_2_O_3_-NPs (50−500 mg/L), however, higher concentrations (1000 mg) had adverse effect on the root structure of soybean [56]. Kulasza et al. [57] described that the root growth of *Setaria italica* was stimulated under 0.8 mg/L Al_2_O_3_-NPs, whereas higher levels (up to 1.8 mg/L) declined the growth of root system. In contrast, CuO-NPs showed toxicity at all tested concentrations in line with the negative responses in similar observational studies in lettuce [58] and spring barley [59]. Cu in nanoform previously showed higher toxicity in comparison to many other metallic NPs, suggesting the high solubility of Cu ions in the plant media. Hussain et al. [60] reported that CuNPs were more toxic than AgNPs and AuNPs to *Artemisia absinthium* plants, inducing the highest inhibitory effect on germination. We also found some evidence that lime plants accumulated an excess amount of Cu and a smaller amount of Al in the plant tissue. Thus, it is likely that the excessive uptake of Cu causes the high toxicity of CuO-NPs compared to Al_2_O_3_-NPs, which may explain our results.

Chlorophylls and carotenoids are distinct photosynthetic pigments that contribute to light absorption and the photosynthetic potential [61]. In the present study, Al_2_O_3_-NPs exhibited a biphasic response in Chl a, Chl b, and CAR, depending on the concentration applied. At 100 mg L⁻¹, Al_2_O_3_-NPs stimulated pigment accumulation in parallel with enhanced biomass production, whereas 200 and 500 mg L⁻¹ caused clear inhibition. This hormetic pattern aligns with Amist et al. [62], who reported enhanced photosynthetic pigments in cabbage at a low concentration (101.8 µM Al_2_O_3_-NPs), followed by a marked decline at higher levels (253.8 µM–2.17 mM). In contrast, CuO-NPs consistently suppressed pigment levels regardless of the concentration applied. Roy et al. [63] observed a similar reduction in maize upon exposure to CuO-NPs, suggesting that excess Cu may substitute Mg in chlorophyll molecules, thereby obstructing energy transfer to PSII. Additionally, excessive ROS generation could impair the photosynthetic apparatus and further reduce pigment content [64]. The contrasting responses to Al_2_O_3_-NPs and CuO-NPs are likely due to differences in their cellular internalization and deposition behavior, driven by distinct structural and chemical properties.

Under nanotoxicity, plants tend to accumulate excessive amounts of ROS, i.e., H_2_O_2_, O2^•−^, and ^•^OH, which instigate lipid peroxidation and impair plant metabolism [65]. The results indicated that 100 and 200 mg/L Al_2_O_3_-NPs did not show toxicity to lime cells. Nonetheless, 500 mg/L Al_2_O_3_-NPs significantly enhanced H_2_O_2_ and lipid peroxidation levels. This could explain the aligning improvements in morphological and photosynthetic parameters under low levels of Al_2_O_3_-NPs while high levels were not favorable to growth and photosynthesis. CuO-NPs, even at the lowest concentration, ominously triggered ROS production. This ROS burst led to cascades of oxidative reactions that increased LOX activity and MG level, the signs of high toxicity [66]. In wheat, Wright et al. [67] illustrated that CuO-NPs had a negative effect on ROS generation and LOX activity, showing membrane dysfunction and redox disturbance. MG, the group of toxic aldehydes, was also increased only under CuO-NPs treatments, suggesting the stronger oxidative stress caused by CuO-NPs compared to Al_2_O_3_-NPs. However, Guo et al. [68] found that the levels of MG were increased in the sensitive Al-treated *Citrus grandis* seedlings but did not change in the Al-tolerant *Citrus sinensis* roots and leaves.

In contrast to CuO-NPs, Al_2_O_3_-NPs elicited specific metabolites in lime tissues (i.e., phenolics, flavonoids, anthocyanins, AsA, and α-tocopherol). These bioactive molecules are among the most important pharmaceutical metabolites in lime that stand out as highly valued medicinal resources [20]. Matching with our observations, Al_2_O_3_-NPs (100–200 mg/L) enhanced the phenolics accrual in sweet basil [13] and was also an efficient elicitor (25–100 mg/mL) for the production of secondary metabolites in *Datura stramonium* [69]. These molecules have significant antioxidative potential thus could support the cellular defense mechanisms and curtail the noxious ROS [66, 70], as we noted in this work. Notably, Al_2_O_3_-NPs stimulated the accumulation of secondary metabolites even at the highest levels, however, increases in H_2_O_2_ and lipid peroxidation were noted at 500 mg/L. This could be attributed to the interference between NPs and diverse signaling pathways like ROS that act as signaling molecules for secondary metabolism. Zhang et al. [71] reported that artemisinin accumulation in *Artemisia annua* following AgNPs exposure was augmented, and this augmentation was accompanied by increased H_2_O_2_ and MDA overproduction, in line with our findings. However, CuO-NPs did not change the pool of phenolics and flavonoids and caused a marked decline in AsA and α-tocopherol, confirming their higher phytotoxicity.

The results attained by the antioxidant analyses indicated that, regardless of the NPs applied concentration, the treatments of Al_2_O_3_-NPs caused a general increase in the activities of SOD, CAT, APX, GPX, GST, PPO, PAL, SPO, and IPO, showing enhanced protective mechanisms. Such antioxidant enzymes play an essential role in mediating potential oxidative damage in stressed plants by acting synergistically to organize the intracellular levels of ROS through different mechanisms [72]. In agreement with our findings, Chahardoli et al. [73] reported enhanced APX, CAT, SOD, and POD activities in *Nigella arvensis* plants treated with Al_2_O_3_-NPs (50–2500 ppm). Ahmed et al. [11] documented a strong correlation between applying nanosized Al_2_O_3_ (0.05–2 mg g^− 1^) and the stimulative activities of CAT and SOD in *Zea mays*. Combining these results with the results of secondary metabolites, Al_2_O_3_-NPs seem to exert a marked impact on the antioxidant system of lime plants, helping them to alleviate oxidative stress in comparison to CuO-NPs, which showed a decline in the activities of SOD, CAT, APX, GPX, GST, and PAL, and an increase in the activities of PPO, SPO, and IPO. These findings were also supported by PCA and heatmap analysis, where a negative correlation was observed for SOD, CAT, APX, and GPX with increasing CuO-NPs concentration. Similarly, Wang and his co-authors [74] reported a marked decline in the activity of SOD, POD, CAT, and APX when *Oryza sativa* plants were subjected to high levels of CuO-NPs (2000 mg/L). This poor defense mechanism reveals that CuO-NPs may pose a potential risk of eliciting oxidative responses in lime compared to Al_2_O_3_-NPs.

Metalloid detoxification molecules, viz., GSH, PCs, and MTCs were also determined in our work. The content of GSH was increased under Al_2_O_3_-NPs and contrarily decreased under CuO-NPs treatments. GSH is one of the central antioxidant metabolites that has a regulatory role in modulating toxicity-induced ROS in the plant tissues by chelating Cu ions in the Haber-Weiss reaction [75]. Owji et al. [76] reported that the contribution of GSH was essential in defending against Al_2_O_3_-NPs in micropropagated fenugreek, which aligns with our results. Moreover, GSH serves as a precursor of important chelation molecules such as PCs and MTCs that are involved in metal-binding homeostasis [77]. Their increases under Al_2_O_3_-NPs treatments through the regulation of corresponding enzymes of GST and GPX may support the advanced defensive network. Moreover, we observed an excessive increase in NO and H_2_S levels in CuO-NPs-treated plants as recently highlighted by Kovács et al. [78]. This intensive increase of NO by CuO-NPs (up to 252% over the control) may reveal the occurrence of nitrosative stress, compared to Al_2_O_3_-NPs (only 30% decrease over control), whereas the high level of H_2_S may be linked to its great accumulation under CuO-NPs. Thus, under Al_2_O_3_-NPs, the increase of NO and H_2_S is still beyond the toxicity level and may act as a signaling molecule in moderating Al_2_O_3_-NPs toxicity. Thereby, the homeostasis balance of ROS-NO-H_2_S is indicative of the stress-tolerant response in Al_2_O_3_-NPs, associated with efficient detoxification of ROS, which was not achieved under CuO-NPs.

The EDX and heatmap analyses reflected a linear correlation between increasing the concentration of NPs in the media and the accumulation of their corresponding ions in the plant tissues. Notably, plants accumulated substantially more Cu than Al. At the highest NP dose (500 mg/L), Cu levels increased by ~ 15-fold relative to the control, compared to only a ~ 2-fold increase in Al. Such intensive accumulation of Cu could be the key basis of CuO-NPs-induced high toxicity to lime cells [79]. It is suggested that the high fraction of Cu was solubilized through complexation with medium salts, enhancing its availability and hence disrupting the uptake of key nutrients such as Mg, Si, S, Cl, P, K, Cu, and Zn. CuO-NPs markedly restricted root development, limiting nutrient absorption and impairing plant growth and cellular metabolism. Some elements were below the detection limit, so they were not reported [80]. Similar findings were reported by Shams et al. [81], who reported reduced N, K, P, Mg, Na, Zn, and B concentrations and non-significant change in other elements under elevated Cu uptake by lettuce. Competitive interference between Cu and Ca, Fe, and Zn at uptake channels may disturb ionic balance and metabolic activity, and growth rate [82]. On the other hand, the excessive accumulation of some elements such as S may not benefit the plants but may cause toxic effects rather than benefit in some cases, as reported by Shahbaz et al. [83] and Hong et al. [84]. On the other hand, Al_2_O_3_-NPs showed different effects on ion profile. Mg increased significantly under 100 mg/L Al_2_O_3_-NPs compared to CuO-NPs, supporting chlorophyll biosynthesis. While disturbance of many elements was noted at higher level (500 mg/L). These results were further validated by PCA and heatmap analysis.

Overall, the percentage increase or decrease of the collected traits under Al_2_O_3_-NPs and CuO-NPs relative to the control plants is presented in Fig. 10, revealing differences in cultivar sensitivity and tolerance to metal oxide NPs. This suggests that the response can vary based on species-specific detoxification pathways and antioxidant defense efficiency. Therefore, lime plants might take advantage of increased production of detoxification enzymes and antioxidant molecules to withstand Cu- and Al-induced stress, even at high NPs’ doses. 

## Conclusion

The increased use of metallic NPs in agricultural and industrial activities could greatly affect the machinery system producing secondary metabolites and antioxidant compounds in plants with medicinal activities. Thus, it is of great concern to study the impact of metallic NPs on their growth and quality traits. Data revealed that Al_2_O_3_-NPs and CuO-NPs showed a wide range of variable effects according to NPs’ type and dose. CuO-NPs stimulated toxicity to lime growth traits, biochemical features, and secondary metabolite production, while Al_2_O_3_-NPs triggered fewer toxic effects and stimulated the generation of secondary metabolites and antioxidant compounds, particularly at the highest concentration (500 mg/L). These variable responses might be associated with the following features: (i) lower Al uptake (~ 2-fold) in plant tissues compared with Cu uptake (~ 15-fold); (ii) the improved capacity of Al_2_O_3_-NPs to eliminate ROS and MG production via regulating secondary metabolites and antioxidant enzymes, thereby supporting sustained growth and photosynthetic capacity, and (iii) increased copper and sulfur accumulation by CuO-NPs that induced toxicity to PCs and MTCs biosynthesis and impairment of NO, H_2_S, and GSH functions, showing hostile oxidative damage and toxic effects. Future research should assess the effects of Al_2_O_3_-NPs and CuO-NPs on lime plants under field conditions to better understand the potential of these NPs for the safe production of medicinally active ingredients. It is also important to consider long-term studies and establish safe threshold levels for different plant species to evaluate the ecological risks of NPs to agroecosystems. This approach will contribute to the development of safe and sustainable nano-agriculture and help control the negative effects on human health and environment.

## Data Availability

All the data are in the published article and will be available upon request.

## Abbreviations

NPs: Nanoparticles
Al_2_O_3_-NPs: Aluminum nanoparticles
CuO-NPs: Copper nanoparticles
APX: Ascorbate peroxidase
Chl a: Chlorophyll a
Chl b: Chlorophyll b
H_2_O_2_: Hydrogen peroxide
O_2_: Superoxide anion
OH: Hydroxyl radical
MDA: Malondialdehyde
MG: Methylglyoxal
CAT: Catalase
LOX: Lipoxygenase
AsA: Ascorbic acid
APX: Ascorbate peroxidase
SOD: Superoxide dismutase
GPX: Glutathione peroxidase
GST: Glutathione-S-transferase
PPO: Polyphenol oxidase
PAL: Phenylalanine ammonia-lyase
SPO: Soluble peroxidase
IPO: Polyphenol oxidase
GSH: Reduced glutathione
PCs: Phytochelatins
MTCs: Metallothionines
NO: Nitric oxide
H_2_S: Hydrogen sulfide
ROS: Reactive oxygen species
PCA: Principal component analysis
